# Atypical Presentation of Metastatic Breast Cancer: Gastric Outlet Obstruction and Linitis Plastica

**DOI:** 10.7759/cureus.80225

**Published:** 2025-03-07

**Authors:** Riley L Smith, Cassidy Ross, Arpankumar Patel

**Affiliations:** 1 Internal Medicine, Liberty University College of Osteopathic Medicine (LUCOM), Lynchburg, USA; 2 Internal Medicine, Kentucky College of Osteopathic Medicine, Pikeville, USA; 3 Internal Medicine, The Christ Hospital, Cincinnati, USA

**Keywords:** breast cancer, case report, endoscopy ercp, gastric biopsy, gastric cancer, gastric outlet obstruction, linitis plastica, nausea and vomiting, vomiting, weight loss

## Abstract

Linitis plastica (LP) is a rare stomach cancer that has a high mortality rate due to late presentation and limited available therapies. LP has a varied clinical presentation and can present at all stages. LP can be a primary or secondary neoplastic process. Rarely, it occurs secondary to breast cancer (BC). In this case report, we present an 88-year-old female patient who has a history of right breast ductal adenocarcinoma. She underwent chemoradiation eight years ago and was in remission when she presented with nausea, vomiting, weakness, and jaundice. During an endoscopic ultrasound and endoscopic retrograde cholangiopancreatography, multiple biopsies were taken of epigastric lymph nodes and the gastric wall. The biopsy showed metastatic BC, and the lymph nodes were positive for metastatic adenocarcinoma, resulting in a diagnosis of LP secondary to BC. Although difficult to diagnose, this case report details the need for a lower threshold of endoscopic evaluation in a patient with a history of BC. It also emphasizes the need for further research into potential treatment options.

## Introduction

Linitis plastica (LP) is a rare type of stomach cancer that can be primary or secondary to another primary neoplastic process. It is called “linitis” due to the microscopic appearance of the neoplasm that looks like a filamentous band of linen and “plastica” due to the hard rubbery appearance of the gastric tissue [1]. Diagnosis is made through an upper endoscopy and biopsy [2]. Breast cancer (BC) is the most common cancer, with an estimated annual incidence of around 310,720, and BC is the fourth leading cause of cancer death in America, with metastasis most commonly seen in the brain and lungs and many other sites [3,4].

LP secondary to BC is very rare; metastatic spread to the gastric tissue happens in less than 10% of patients with BC [5]. It is clinically silent until proven by a biopsy. The prevalence is largely unknown and will require additional research [4]. Histology varies depending on the infiltrating neoplasm, whether it is primarily gastric or secondary to breast metastasis. The clinical presentation varies greatly depending on the advancement of primary or secondary neoplastic processes, and managing this disease is usually difficult. We present an interesting case of LP secondary to BC in an elderly woman.

## Case presentation

An 88-year-old female patient with a history of estrogen receptor (ER)-positive, progesterone receptor (PR)-negative, and human epidermal growth factor receptor 2 (HER2)/neu-negative right ductal breast adenocarcinoma believed to be in remission and prior chemoradiation eight years ago presented with complaints of nausea, vomiting, and weakness of two weeks' duration. On physical examination, she appeared jaundiced on initial presentation, and the laboratory results revealed moderately elevated liver enzymes, alkaline phosphatase, and total bilirubin levels three times the normal range with direct hyperbilirubinemia, as shown in Table 1. Due to the hyperbilirubinemia, a gastric stent was placed to allow passage of the gastric contents while that patient received IV fluids and bilirubin binding agents. These fluids and binding agents allowed time for the stent to be placed and gave the patient time to decide if the patient would want palliative care, surgery with chemotherapy, or to be admitted to a hospice service. The patient ultimately elected to be admitted to hospice service following stent placement.

**Table 1:** Initial patient lab values

Laboratory parameters	Initial patient values	Normal ranges
Alanine transaminase	146	0-40 U/L
Aspartate transaminase	149	0-30 U/L
Alkaline phosphatase	553	33-140 U/L
Total bilirubin	3.6	0.2-1.2 mg/dL
Direct bilirubin	3.9	0-0.5 mg/dL

**Figure 1:**
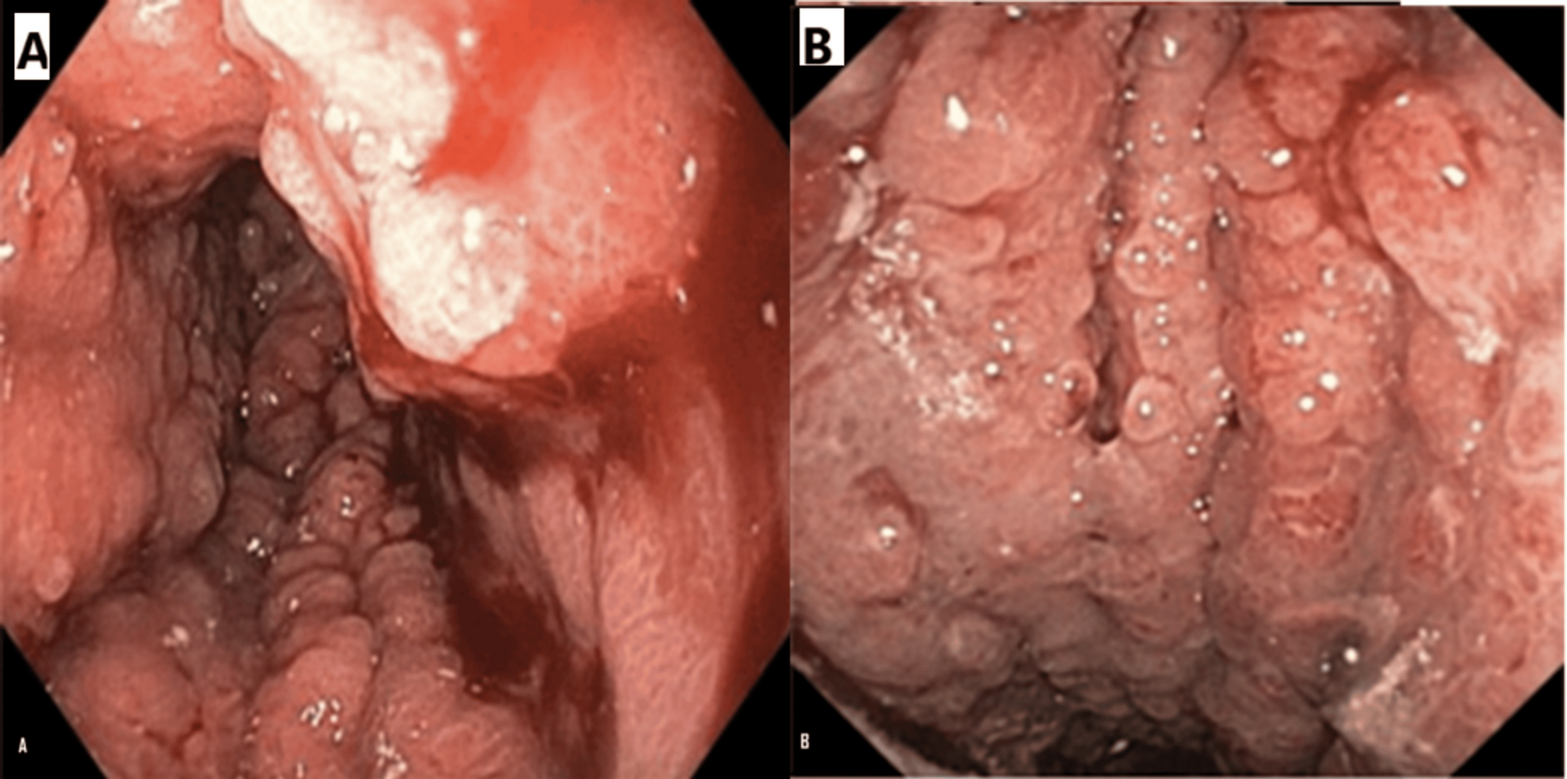
Photographs from attempted ERCP. (A) The irregular knotted wool-like appearance. (B) Irregularity of the tubular processes and the hypertrophy of the gastric folds ERCP: endoscopic retrograde cholangiography

Evaluation with endoscopic retrograde cholangiography (ERCP) failed because the scope could not be advanced beyond the duodenal bulb due to a visibly suspected neoplastic process. Endoscopic ultrasound noted multiple perigastric lymphadenopathy, and samples were collected for histopathological evaluation. Endoscopic evaluation of the gastric antrum noted significant thickening, easy bleeding, and inflammation, as seen in Figure 1. The endoscopic images display the thickening of gastric mucosa with a leathery appearance. It was also noted that the ERCP multiple biopsies were taken from the gastric wall and perigastric lymph nodes. The biopsy results can be seen in Table 2. The results of this biopsy are initially surprising if suspected to be a metastasis from the primary cancer believed to be in remission. However, metastases are known to sometimes have different immunohistochemical staining than the primary tumor [6]. This could be a possible explanation for the discordance between the history of BC and the new neoplastic process.

**Table 2:** Comparison of metastatic tumor findings between perigastric lymph nodes and gastric biopsies Ki-67: a marker of actively dividing cells; ER: estrogen receptor; PR: progesterone receptor; HER-2-neu: human epidermal growth factor receptor 2 Source: [7-9]

Test	Perigastric lymph nodes	Gastric wall	Reference ranges
Ki-67	60%	60%	Below 5% for lymph nodes and gastric tissue
ER	<1%	<1%	Less than 1%
PR	Negative	Negative	Negative
HER-2-neu	Zero	Zero	0 to +3

**Figure 2:**
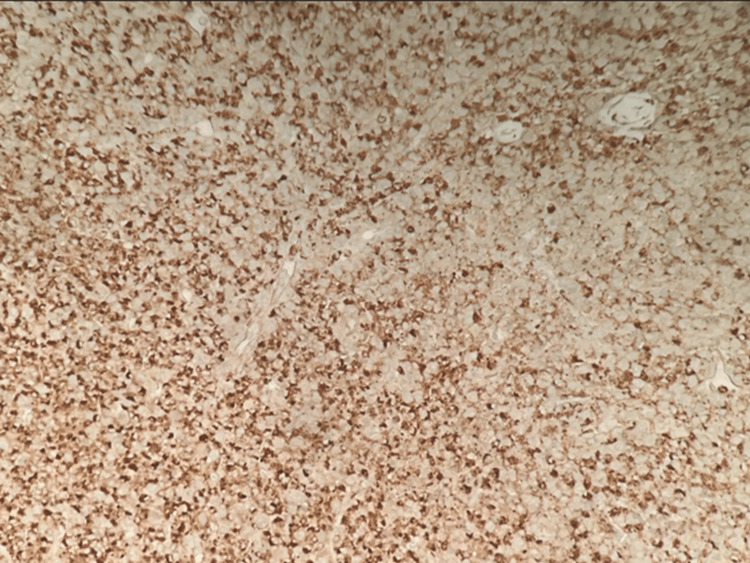
Biopsy from gastric tissue histopathology The gastric mucosa is infiltrated by atypical epithelial cells in small nests and singly. The tumor cells have eosinophilic cytoplasm and round nuclei with irregular nuclear membranes, hyperchromasia, and small nucleoli. The tumor is GATA-3-positive, supporting a breast primary. ER, PR, and HER2 are negative GATA-3: GATA binding protein-3; ER: estrogen receptor; PR: progesterone receptor; HER2: human epidermal growth factor receptor 2

The gastric mucosa is infiltrated by atypical epithelial cells in small nests and singly. The tumor cells have eosinophilic cytoplasm and round nuclei with irregular nuclear membranes, hyperchromasia, and small nucleoli. The tumor is GATA binding protein-3 (GATA-3) positive, supporting a breast primary. ER, PR, and HER2 are negative (Figure 2).

## Discussion

LP is characterized by distinctive histopathologic features, where malignant cells infiltrate the gastric mucosa, causing the stereotypical appearance of the gastric mucosa seen on ERCP. To determine if the etiology is from primary gastric cancer or secondary metastatic disease, a biopsy must be performed. The hallmark feature of LP is diffuse mucosal wall infiltration, particularly affecting the submucosa and muscularis propria layers [1]. This infiltrative process leads to characteristic physiological changes, including decreased gastric compliance and volume, which manifests clinically as gastrointestinal symptoms. Patients typically present with nausea, vomiting, poor appetite, weight loss, and anorexia, often complicated by gastric outlet obstruction [10].

The diagnosis of LP presents significant challenges due to the limitations of current biopsy techniques and the indistinct border between neoplastic and normal gastric tissue. Initial biopsies frequently yield negative or nonneoplastic results, necessitating fine-needle aspiration and multiple biopsy attempts to obtain sufficient diagnostic tissue [2]. This can be seen in a few case reports. While the complete pathogenesis of LP remains unclear, several genes have been implicated in its development, including human epidermal growth factor-2 (HER2) and E-cadherin [11], which play crucial roles in vascular invasion and tumor growth. The tumor microenvironment can be a source of difference in hormonal receptor statuses between the previous primary BC and the new metastasis to the gastric tissue [12]. These other case reports have shown that one clear unifying factor is gastric outlet obstruction and that the hormonal status of the original BC was not the same [13,14]. The prognosis is notably poor regardless of treatment approach [15], with survival rates averaging 16.7 months for surgical patients and merely 3.6 months for those without surgical intervention [16]. Our patient exemplified the complexity of secondary LP, presenting with ER-positive, PR-negative, and HER2/neu-negative right ductal breast adenocarcinoma metastases in an advanced stage. The management of LP poses particular challenges due to its varied presentations and the lack of consensus guidelines for treatment approaches, especially in cases of secondary origin. Patients with secondary LP often present with multiple metastatic sites [17], leading to predominantly palliative treatment strategies. A recent case series pointed out that there was no real difference in surgical and palliative treatment courses, but the article also highlights how rare of a disease this is compared to primary gastric cancer with the case series only containing 12 patients [18]. The disease burden significantly impacts the quality of life through multiple mechanisms: gastric outlet obstruction, severe cachexia, malabsorption from decreased acid secretion, and limited gastric motility [1].

In our case, the constellation of findings, including the patient's age, endoscopic appearance, and symptoms of gastric outlet obstruction strongly suggested LP, which was subsequently confirmed by biopsy. The endoscopic images (Figure 1) demonstrated the characteristic waffle-like or hypertrophic gastric walls typical of LP [19]. The differential diagnosis for LP extends beyond primary gastric carcinoma to include various malignancies (lymphoma, bladder, colonic, and gallbladder cancers) and nonneoplastic conditions (caustic ingestion, tuberculosis, gastric amyloidosis, and sarcoidosis) [20-22]. Given the macroscopic similarities among these conditions on endoscopy, definitive diagnosis relies critically on histopathology and immunohistochemistry. The universally poor outcomes, regardless of treatment approach, reflect the advanced stage at which most cases are diagnosed.

## Conclusions

LP secondary to BC, while rare, carries a significant mortality rate primarily due to delayed diagnosis and limited treatment options, as demonstrated in our case of an elderly patient with a history of treated BC. Although our patient presented with classic symptoms of gastric outlet obstruction, the diagnosis of LP required multiple biopsies and detailed immunohistochemical analysis. This case emphasizes the importance of maintaining a high clinical suspicion for gastric metastases in patients with a history of BC who present with persistent gastrointestinal symptoms, particularly when endoscopic findings reveal characteristic thickening and rigidity of the gastric wall. The challenging diagnosis in our patient, despite her previous BC history, highlights the need for a lower threshold for endoscopic evaluation and biopsy in similar cases. Moving forward, research efforts should focus on several key areas: identifying nongenetic risk factors, developing systematic algorithms to guide the timing of endoscopic evaluation, and exploring therapeutic options beyond the current standard of palliative chemotherapy and surgery. Understanding these aspects could potentially improve both the early detection and treatment outcomes for patients with this challenging condition.
